# A Conservative Approach to a Peripheral Ameloblastoma

**DOI:** 10.1155/2016/8254571

**Published:** 2016-10-20

**Authors:** Rocco Borrello, Elia Bettio, Christian Bacci, Marialuisa Valente, Stefano Sivolella, Sergio Mazzoleni, Mario Berengo

**Affiliations:** ^1^Section of Dentistry, Department of Neurosciences, University of Padova, Padova, Italy; ^2^Department of Cardiac Thoracic and Vascular Sciences, University of Padova, Padova, Italy

## Abstract

Peripheral Ameloblastoma (PA) is the rarest variant of ameloblastoma. It differs from the other subtypes of ameloblastoma in its localization: it arises in the soft tissues of the oral cavity coating the tooth bearing bones. Generally, it manifests nonaggressive behavior and it can be treated with complete removal by local conservative excision. In this study we report a case of PA of the maxilla in a 78-year-old female patient and we describe the four different histopathological patterns revealed by histological examination. After local excision and diagnosis, we planned a long term follow-up: in one year no recurrence had been reported. The choice of treatment is illustrated in Discussion.

## 1. Introduction

Ameloblastoma is a benign odontogenic tumour which originates from ameloblasts. It commonly arises between the third and the fourth decade of life [1–3] and it can occur either in the jawbones or in the gingival soft tissue. There are three subtypes of ameloblastoma: solid and multicystic ameloblastoma, unicystic ameloblastoma, and Peripheral Ameloblastoma (PA) [4, 5]. The first two subtypes are localized in the bone tissue of maxilla and mandible; both are locally aggressive tumours with recurrence potential. PA, also known as extraosseous ameloblastoma, is an extremely rare variant, representing 1-2% of all ameloblastomas [6]. It develops in the gingiva [3, 7, 8], usually in the area of mandibular canine/premolar [7], and can be clinically observed as an exophytic sessile nodule with firm consistency [4, 8]. It is generally painless and nonradiolucent and it is thought to derive from the gingival epithelium or from remnants of the dental lamina [8, 9].

## 2. Case Report

**Figure 1:**
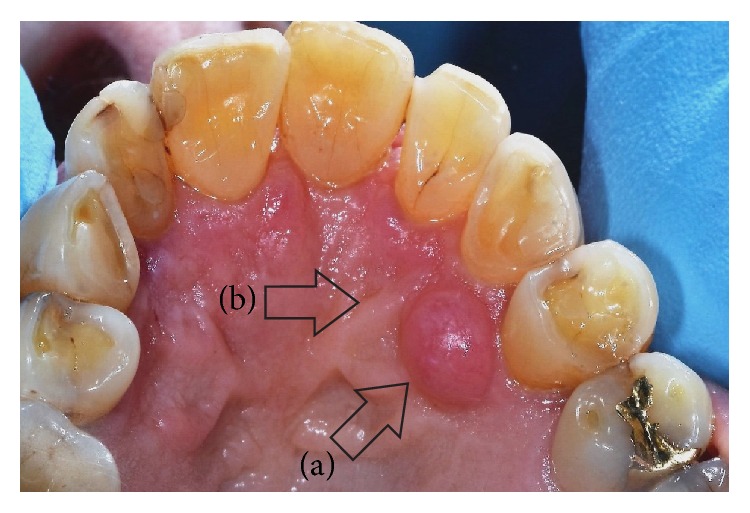
Clinical aspect of the PA. (a) The main lesion. (b) The smaller lesion.

**Figure 2:**
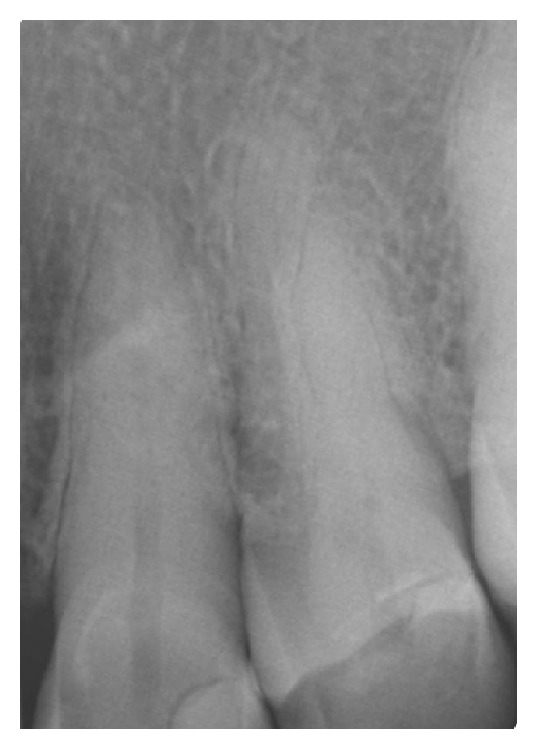
Intraoral radiograph of the PA.

**Figure 3:**
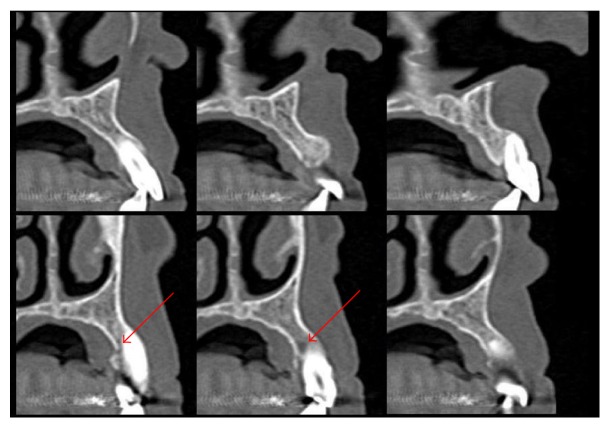
Preoperative CT. The red arrows point to the small depression of the bone surface in correspondence of the tumour (“cupping” or “saucerization”).

A 78-year-old female patient was referred to the Dental Clinic of the University of Padua in order to evaluate a painless swelling on the palatal mucosa located near the superior left canine. The lesion, as described by the patient, was first noticed 10 years before. The patient was a nonsmoker and was under treatment for hypertension (with ACE inhibitor, beta blocker, and low-dose aspirin). Oral examination revealed two adjacent lesions, covered with normal coloured mucosa, measuring 7 × 5 × 5 mm and 4 × 3 × 3 mm (Figure 1). The main lesion appeared as a hard painless gingival swelling with smooth surface; the smaller one was a pedunculated outgrowth of the palatal mucosa with soft consistency. No associated lymphadenopathy was detected. X-ray examinations (intraoral radiograph and computed tomography) showed slight bone resorption in correspondence of the lesion (Figures 2 and 3).

**Figure 4:**
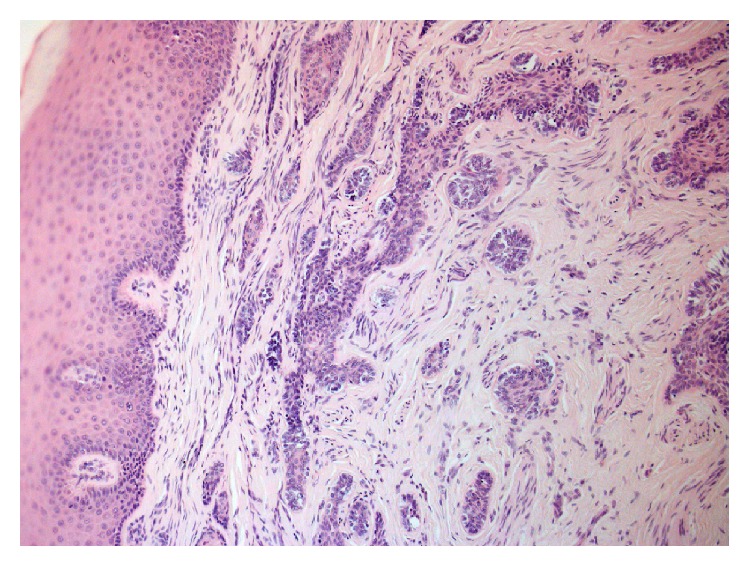
Histological aspect of the PA.

**Figure 5:**
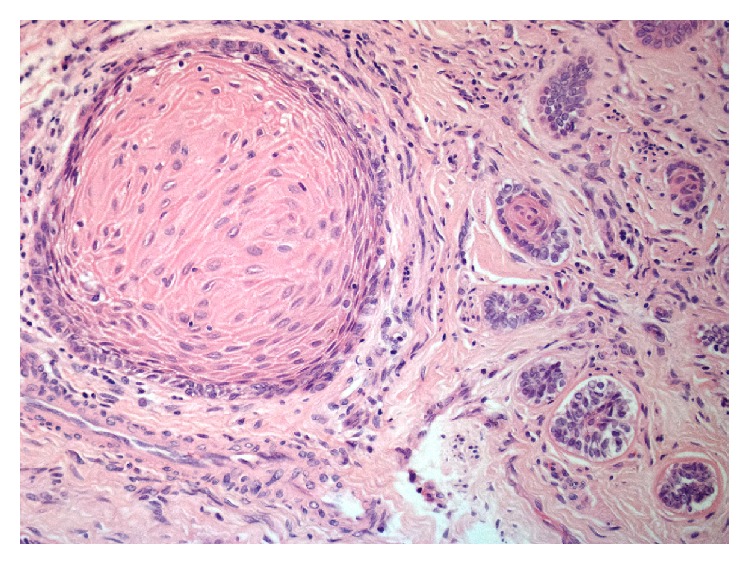
Follicular and acanthomatous cell patterns.

**Figure 6:**
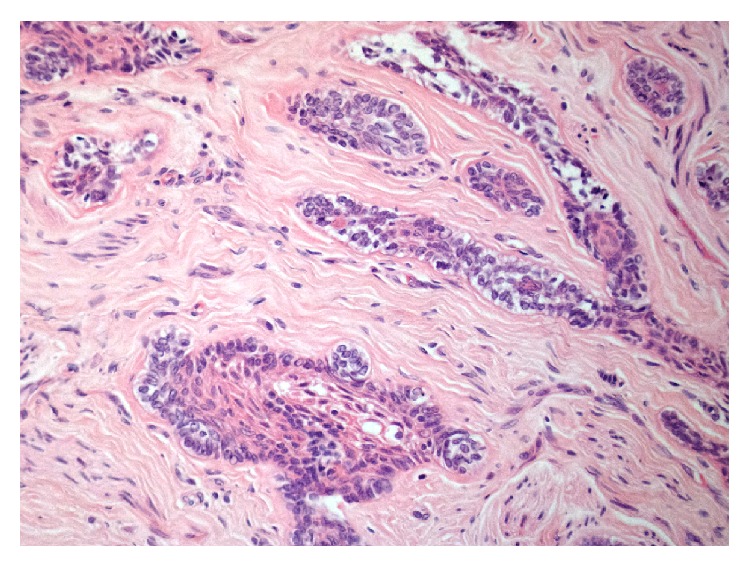
Plexiform pattern.

**Figure 7:**
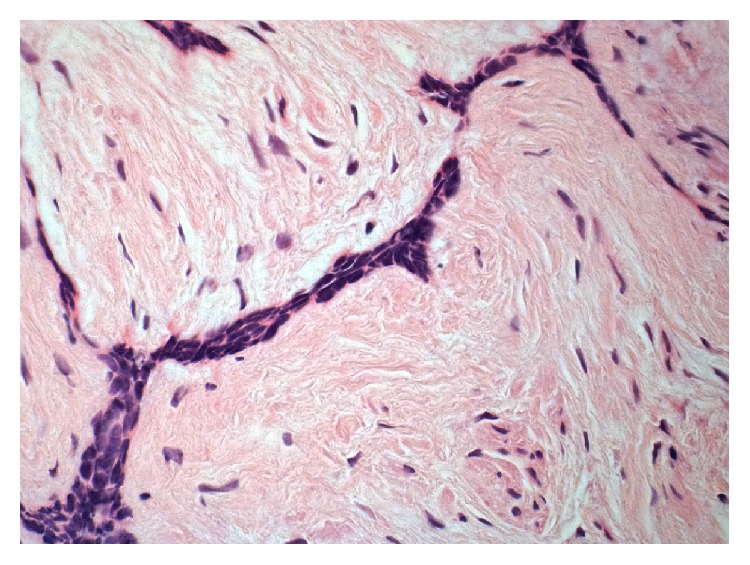
Desmoplastic pattern.

Excisional biopsy of the two lesions was performed under local anaesthesia, and the tissues were submitted to histopathological examination. Microscopic examination of the main lesion showed a mucosal mass covered by stratified squamous epithelium (Figure 4). The lamina propria contained multiple cords and small islands of epithelial tumour cells with ameloblastic features. The peripheral tumour cells often exhibited hyperchromatic, columnar nuclei with a palisaded arrangement and areas of reverse nuclear polarity. In some areas, the cells of the tumour islands showed an acanthomatous pattern with central squamous differentiation (Figure 5, left side). Other parts of the lesion consisted of narrow ribbon-like cords that were suggestive of an early plexiform pattern (Figure 6). The minor lesion also revealed the presence of tumour cells with a desmoplastic pattern, consisting of thin cords (only a few cells in width) of odontogenic epithelium dispersed in a dense collagenous stroma (Figure 7).

**Figure 8:**
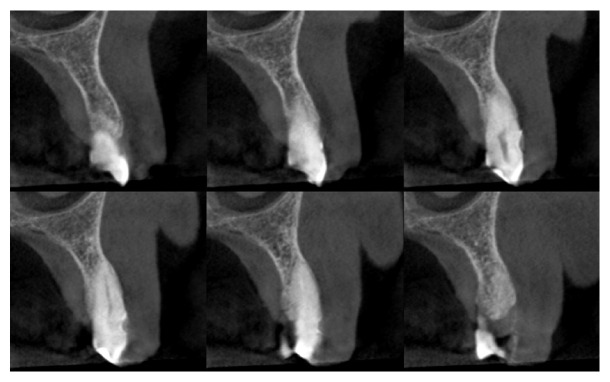
CT 10 months after biopsy.

**Figure 9:**
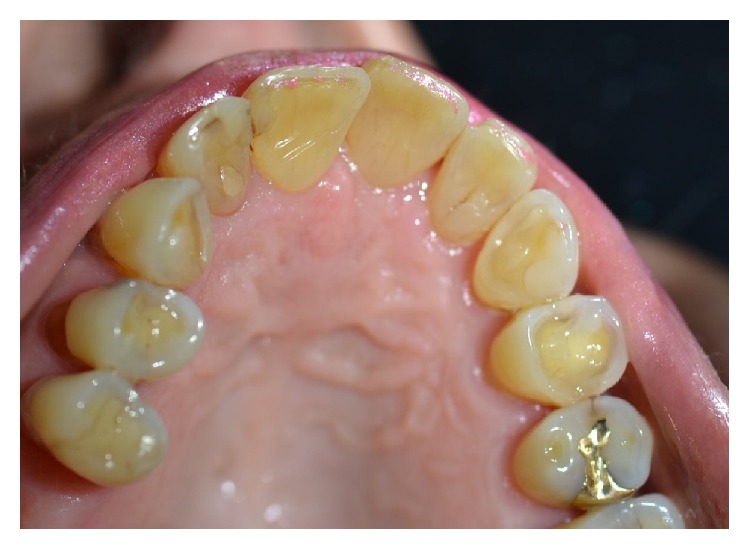
Palate clinical view 1 year after biopsy.

According to the clinical, radiographic, and histopathological exams, a PA was diagnosed. Four months after the excisional biopsy the surgical wound appeared healed by secondary intention. Further surgical approach (a radical resection) was deemed unnecessary and a two-month follow-up was planned. After two months the lesion area was clinically unchanged. A second CT performed 10 months later did not show the superficial bone resorption, confirming the tumour was not infiltrating the bone (Figure 8). After one year no relevant clinical alteration could be observed (Figure 9).

## 3. Discussion

Treatment of the subtypes of ameloblastoma is still controversial and it is based on recurrence potential and aggressivity of each subtype. In addition, the choice of treatment depends not only on the apparent microscopic pattern on biopsy, but also on the tumour location, size of the lesion, age of the patient, and reliance of the patient for good long term follow-up.

Whereas radiotherapy and chemotherapy are not recommended techniques, surgical intervention, radical or conservative resection, is the preferred management for ameloblastomas [2].

Radical resection can be marginal or segmental and it is associated with a recurrence rate ranging from 0% to 10% [2, 4]. Such a treatment can be beneficial in maxillary ameloblastoma (which acts clinically more aggressively for the lack of the thick cortical bone found in mandible that can slow down the tumour growth) [2].

Solid and multicystic ameloblastoma can be treated with a surgical approach extended up to 1–1.5 cm around radiographic or histologic margins of the lesion since, because of its capacity of infiltrating the bone, it is regarded as a locally aggressive tumour [2, 4]; conservative resection, such as curettage and enucleation, is associated with a high recurrence rate (solid and multicystic 60–80%, unicystic 30–60%) [2–5, 10–19]; for this reason it can be paired with cryotherapy, electrocautery, or tissue fixatives like Carnoy's solution [2].

Unicystic ameloblastoma, which is thought to be less aggressive than solid or multicystic ameloblastoma, often can be treated with enucleation and peripheral ostectomy sometimes supplemented by physicochemical treatment (cryotherapy, electrocautery, or tissue fixatives) [4]. However, some cases may require more aggressive surgical resection to be performed [4, 20].

PA, per contra, manifests a benign behaviour with an average growth rate lower than other subtypes of ameloblastoma (0.17 versus 0.81 cm^3^/month, resp.) [2, 21]. Moreover, bone involvement of PA is absent or irrelevant, appearing as a small depression of the bone surface in correspondence of the tumour (named “cupping” or “saucerization”) (Figure 3) [3–5, 8, 9]. The surgical treatment of choice for PA consists in conservative local excision without removing bone or teeth [2, 4, 8, 9, 13].

In the reported case the tumour appeared as an exophytic sessile lesion and the patient had no symptoms. Intraoral examination as well as the radiographs gave no useful indication in order to formulate the correct diagnosis. The lesion was treated with local excision and, after the response of the histopathologic laboratory, further surgical approaches were judged as unnecessary overtreatments. Nevertheless, considering the recurrence rate of the PA (from 16% to 19%) [8, 22, 23] a long term follow-up was planned with the purpose of detecting any recurrence which could possibly develop in the future.
